# Phylogeny of *Paecilomyces*, the causal agent of pistachio and some other trees dieback disease in Iran

**DOI:** 10.1371/journal.pone.0200794

**Published:** 2018-07-24

**Authors:** Reza Heidarian, Khalil-Berdi Fotouhifar, Alfons J. M. Debets, Duur K. Aanen

**Affiliations:** 1 Department of Plant Protection, Faculty of Agricultural Science and Engineering, College of Agriculture and Natural Resources, University of Tehran, Karaj, Iran; 2 Laboratory of Genetics, Plant Sciences Department, Healthy Food and Living Environment Faculty, Wageningen University, Wageningen, The Netherlands; Leibniz Institut - Deutsche Sammlung von Mikroorganismen und Zellkulturen GmbH, GERMANY

## Abstract

One of the most important fungal agents of pistachio dieback disease belongs to the ascomycete genus *Paecilomyces* that has been identified as *P*. *variotii*. In 2012–2014, 700 plant samples from pistachio trees and 27 other plant species with dieback symptoms were collected from 10 provinces of Iran. Of the 567 pistachio samples, 277 *Paecilomyces* strains were obtained and from the 133 samples of other plants (except pistachio and including *Pistacia mutica*, *Punica granatum*, *Prunus amygdalus*, *Caesalpinia gilliesii*, *Nerium oleander*, *Tamarix aphylla*, *Tamarix hispida* and *Haloxylon* sp.), 23 fungal isolates were recovered and five isolates were obtained from the air of infected pistachio orchards. Based on morphology, all 305 isolates were identified as *P*. *variotii*. Physiological studies revealed that 299 isolates belong to *P*. *formosus*. Three isolates were assigned to *P*. *variotii*, while three isolates could not be assigned to any of the known species. Of the 305 isolates, 62 were selected for phylogenetic analysis based on DNA variation (*ITS*, *β-tubulin* and *calmodulin*). This analysis showed that all of our isolates form a clade with *P*. *formosus*. *P*. *formosus* consists of the three former species *P*. *formosa*, *P*. *lecythidis* and *P*. *maximus*. This study shows that our isolates form a strongly supported clade with strains of *P*. *lecythidis*. So, the causal agent of dieback disease of pistachio and other examined trees is *P*. *formosus* which is closely related to the former species *P*. *lecythidis* and has some differences with the former species *P*. *formosa* and *P*. *maximus*. Based on phylogenetic studies *P*. *formosus* thus seems to be a species complex that could be divided into three separate species.

## Introduction

Iran is a one of the largest producers and exporters of pistachio in the world. Pistachio dieback is a major disease in Iranian pistachio orchards, infecting most of the host varieties that grow under unfavorable conditions [1, 2]. Symptoms of dieback disease are cluster, leaf, and bud wilting, necrosis and darkening of bark and wood, canker and finally dieback. Cankers begin at wounds or dead areas in the bark of the stems or twigs, and expand in all directions from the point of infection but grow much faster along the main axis of the stem, branch, or twig and develop in the bark and the wood. The color of infected tissues changes to dark brown to black. Cankers are often sunken beneath the surface of the bark and ooze gum. In some cankers, the healthy tissues immediately next to the canker may increase in thickness and appear higher than the normal surface of the branch as a result of producing callus tissue around the infected areas. The bark in the cankered area is roughened and cracked. If the canker girdles the twig, branch or stem, all tissues higher than the canker will wilt and die [2–5].

Historically, Bainier [6] introduced the genus *Paecilomyces* as a closely related taxon to genus *Penicillium* Link ex Fr., but it has been indicated that *Paecilomyces* differs from *Penicillium* by the absence of green-coloured colonies and by the shape of phialides which have a cylindrical basal portion and a long neck.

Brown and Smith [7] provided a comprehensive monograph of the genus *Paecilomyces* and in that treatment of the genus, they transferred some species of *Isaria* and *Spicaria* to *Paecilomyces*, which at that moment contained 23 species, based on similar conidiogenous structures as seen in *Paecilomyces variotii*.

Samson [8] also monographed and redefined the genus *Paecilomyces* (type species: *P*. *variotii* Bainier) based on morphological characteristics, the perfect states that belonged to genera *Byssochlamys* Westl., *Talaromyces* C. R. Benjamin and *Thermoascus* Miehe. Species of the genus *Paecilomyces* are identified by having verticillate conidiophores bearing divergent whorls of branches and phialides. The phialides are cylindrical or inflated at the base, which taper to a long distinct neck and produce basipetal chains of single-celled hyaline conidia. The genus was sub-divided into two sections, sect. *Paecilomyces*, including some mesophilic to thermophilic species having yellow-brown to brown colonies and several species accompanying with perfect state and sect. *Isarioidea*, including mesophilic species with white or other bright colonies. Totally, 31 species were described and illustrated based on their morphological characteristics produced in pure culture.

Luangsa-ard and Hywel-Jones [9] used *18S rDNA* sequence data for phylogenetic analysis of the genus *Paecilomyces* and demonstrated that the genus is polyphyletic across the Sordariomycetidae and Eurotiomycetidae and indicated the morphological variation of the type species, *P*. *variotii*.

Based on micro- and macroscopical investigations of *Byssochlamys* and *Paecilomyces variotii*-like isolates, Houbraken et al. [10] showed that the genus *Byssochlamys* and its related anamorphic species can be divided into at least nine taxa.

By adding molecular data and extrolite profiles, Samson et al. [11] extended this to a multiple evidence approach and provided a revised taxonomy and nomenclature of the accepted taxa and mentioned that the genus *Paecilomyces* is only monophyletic taxon within the order Eurotiales that is characterized by a *Byssochlamys* teleomorph. The *P*. *variotii* species complex could be divided into five species, *P*. *divaricatus*, *P*. *formosus*, *P*. *brunneolus*, *P*. *dactylethromorphus* and *Byssochlamys spectabilis* (the sexual state of *P*. *variotii*) [11–12]. *P*. *formosus* may be composed of three distinct species, including *P*. *formosus*, *P*. *lecythidis* and *P*. *maximus*. However, these three taxa could be distinguished only by molecular phylogeny data and not by microscopical characters and the extrolite analyses. One distinction between isolates of ‘*P*. *maximus*-clade’ and the other members of this diverse group is the faster growth rate of this species at 37°C than at 30°C. [11]. *ITS* regions and part of the *β-tubulin* gene were shown to exhibit sufficient inter-specific variation for identification of *Paecilomyces* strains in clinical samples. *P*. *variotii*, *P*. *formosus*, *P*. *dactylethromorphus*, and *P*. *divaricatus* have the similar antifungal susceptibility profiles [12].

Alizadeh et al. [3] studied the etiology of the causal agent of the dieback disease in pistachio trees in Iran and introduced it as *Paecilomyces variotii*. Gelichi et al. [5] also confirmed the pathogenicity of several isolates of *Paecilomyces* on pistachio in vitro and in situ and introduced them as *Paecilomyces variotii*. Two species of the genus *Paecilomyces* have been reported from different hosts from Iran including: *P*. *variotii* from almond, pistachio and sesame [13] and *P*. *tenuis* from wheat [14].

The mycelium of these fungi overwinters on the infected parts. Most infections take place in late fall or early winter. The fungus enters through wounds of injuries or leaf and cluster scars [2–5]. Heidarian et al. [15] studied the mating type idiomorphs and sexual reproduction possibility of *Paecilomyces formosus* in vitro. They designed two primer pairs including; Mat1-1f224 and Mat1-1r224 for amplification of *Mat1-1* and Mat1-2f165 and Mat1-2r165 primers for amplification of *Mat1-2* idiomorphs. Mating type idiomorphs were amplified for 124 isolates of *P*. *formosus*. In 50 isolates (40.3%) *Mat1-1*, in 59 isolates (47.6%) *Mat1-2* and in 15 isolates (12.1%) both idiomorphs were detected. 16 isolates from each mating type and three isolates which had both idiomorphs were selected and crossed with each other in all of possible combinations. After eight months, sexual reproduction was not observed in all crosses.

Until now, all researchers in Iran have used the monograph of Samson [8] for the identification of *Paecilomyces* species based on morphology. Therefore, *P*. *variotii* has been reported as the only causal agent of dieback disease on *Pistacia vera* in Kerman and Khorasan-Razavi provinces in Iran [2–5] and on almond in Shahrekord [16]. The aim of this research was to identify the *Paecilomyces* strains associated with dieback disease of pistachio based on morphology, physiology and molecular phylogeny using *ITS*, *β-tubulin* and *calmodulin* sequences, and to study host specificity, geographic distribution and geographic differentiation in Iran.

## Results

### Samples & fungal isolates

At least 85% of inspected pistachio orchards had disease symptoms, 1–100% of pistachio plants in affected orchards were showing symptoms of die-back and 1–100% (average 35%) of branches in infected trees had been injured by die-back disease. We obtained 700 plant samples of which 567 were from pistachio trees and 133 from 27 other plant species growing in pistachio cultivation areas. Of the 567 pistachio samples, 277 *Paecilomyces* strains were isolated. Of the 133 samples that were collected from the other 27 plant species, 23 fungal isolates were recovered from only nine of non-pistachio hosts. Furthermore, five isolates were obtained from the air of infected pistachio orchards (Table 1).

**Table 1:** Obtained isolates of *Paecilomyces*.

Host	Common name	No. of isolates	Year	Location (province)
*Pistacia vera*	Pistachio	277	2012, 2013, 2014	All of 10 provinces
*Pistacia mutica*	Wild pistachio	2	2013	Kerman
*Punica granatum*	Pomegranate	5	2012,2013	Kerman, Yazd, Khorasan-Razavi
*Prunus amygdalus*	Almond	3	2013	Yazd, Khorasan-Razavi
*Caesalpinia gilliesii*	Bird of paradise	1	2014	Kerman
*Haloxylon *sp.	Saxaul	4	2013	Kerman, Khorasan-Razavi
*Nerium oleander*	Oleander	1	2013	Yazd
*Tamarix aphylla*	Salt cedar	2	2013	Kerman, Khorasan-Jonubi
*Tamarix hispida*		5	2013	Semnan, Kerman, Yazd, Khorasan-Razavi
The air of orchards		5	2013	Kerman

### Morphological and physiological studies

The morphology and physiology of all of 305 isolates were studied. The colony color on malt extract agar (MEA, 48Gr/L) was variable among the strains, ranging from yellowish cream, olive cream to olive. With age, colonies changed to darker shades. The bottom of Petri petri dishes with the colonies varied from cream to olive, brown and black. Vegetative hyphae were hyaline, mostly thick-walled, 2–6 μm wide. Conidiophores consisted of verticillate or irregular branches, each branches have 2 to 7 smooth-walled phialides. Phialides have an ellipsoidal to cylindrical basal portion, tapering into a long cylindrical, 1.0–2.0 μm (Av. = 1.01, σ = 0.1023) wide neck. Phialides were variable in size, mostly 8–20 (Av. = 12.266, σ = 2.3434)×2–3 (Av. = 2.1858, σ = 0.38) μm. Conidia were sub-globose to ellipsoidal with truncate ends, 3.5–9 (Av. = 4.77, σ = 0.7925)×1–3.5 (Av. = 2.04, σ = 0.21067) μm. Chlamydospores were present, brown or dark brown, smooth-walled, globose, sub-globose to pyriform, thick-walled, 3–11 (Av. = 5.32, σ = 1.2667) μm in diameter. The perfect stage or ascomata initials were not observed in purified isolates.

At first, Samson's monograph [8] was used to show that our isolates are exactly identical to the strains which previously had been identified as *Paecilomyces variotii* and reported as causal agent of pistachio and almond dieback in Iran. Then, morphological, physiological and phylogenetic studies were used to detailed identification of the isolates.

With respect to the morphology, all of 305 isolates could be assigned to *Paecilomyces variotii* sensu lato in agreement with morphological studies by Samson [8]. Absence of ascigerous state or ascoma initials divides *P*. *variotii* from *P. niveus, P. zollerniae, P. crustaceus, P. byssochlamydoides* and *P. leycettanus*. Simple conidiophores, conidia which are surrounded by a mucilaginous layer and green color of reverse of colony were separated *P. aerugineus* from *P. variotii* and *P. clavisporus. P. variotii* with repeatedly branched conidiophores and different size and shape of conidia (mostly sub-globose to broadly ellipsoidal) was segregated from *P. clavisporus* that have simple conidiophores and long cylindrical to clavate conidia [8], but *Paecilomyces variotii* sensu lato have been splited into five species based on polyphasic taxonomy [11].

**Fig 1:**
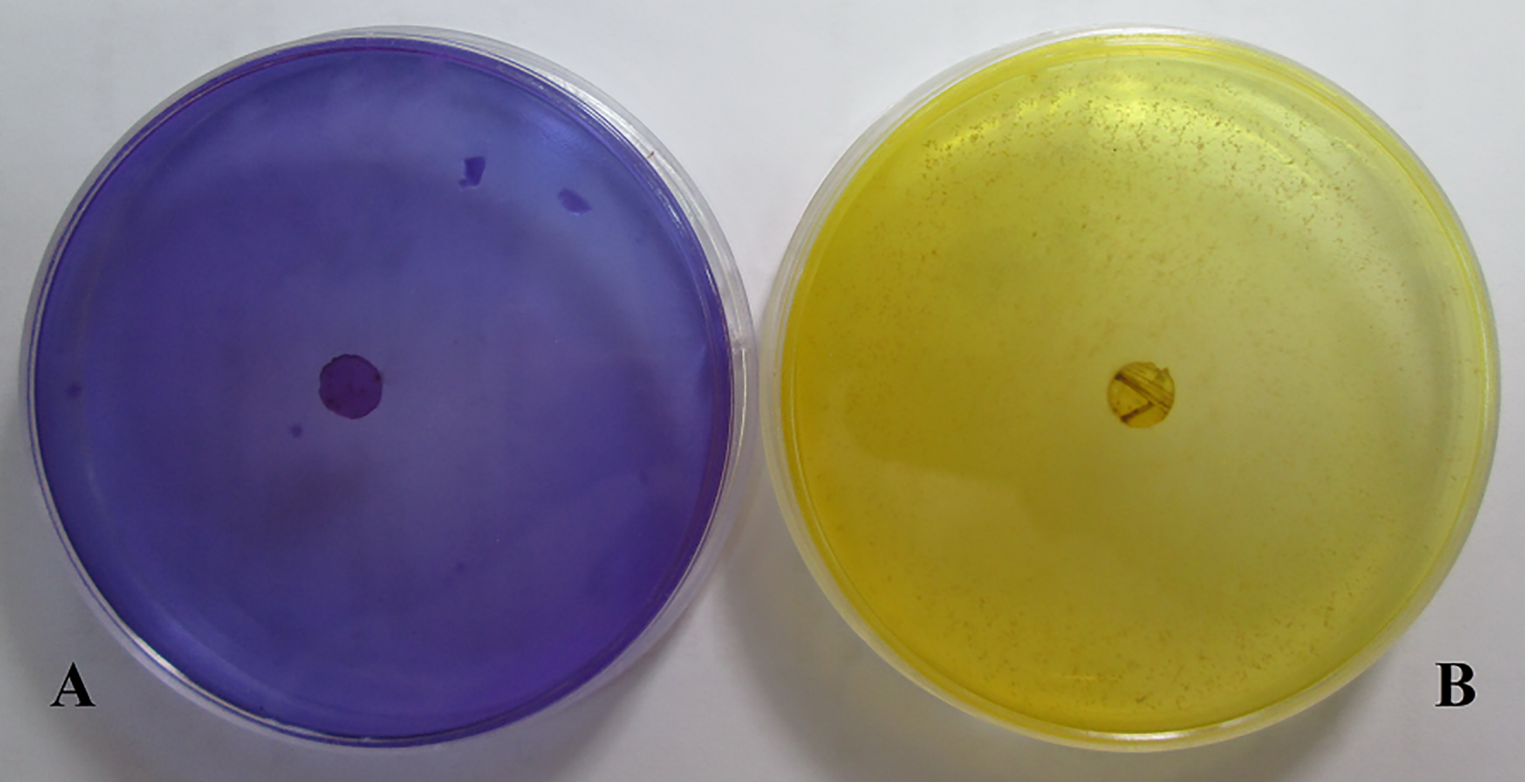
Acid production on CREA. (A) Creatine sucrose agar (CREA) plate inoculated with isolate Pf623 which was unable to grow and produce acid, hence an unchanged purple plate color and (B) CREA plate inoculated with isolate Pf613 which was able to grow and produce acid at 25°C for seven days in continuous dark condition; acid production indicated by the color change to yellow.

All isolates grew at 25, 30 and 37°C on MEA and Czapek yeast autolysate agar (CYA). Colonies grew rapidly on MEA, attaining a diameter more than 45 mm within seven days at 30°C and continuous dark condition (average 72 mm/week) for 302 isolates. Three isolates, including Pf108, Pf391 and Pf804, grew less than 45 mm per week. 302 isolates could grow and produce acid on creatine sucrose agar (CREA) (Fig 1) but three isolates, including Pf518, Pf623 and Pf628, did not. Based on morphological and physiological studies, we concluded that 299 isolates belong to *P*. *formosus* (Sakag., May., Inoue and Tada) Houbraken and Samson, corresponding to Samson et al. [11]. The three isolates which could not grow or produce acid on CREA could be assigned to *P*. *variotii*. Three isolates with a low growth rate on MEA at 30°C, produced chlamydospores and acids on CREA. The ratio of growth rates at 37 to 30°C on MEA was 0.6, 1 and 1.3 for strains Pf108, Pf391 and Pf804, respectively. Therefore, these strains could not be assigned to any known species based on the classification of Samson et al. [11].

### Phylogenetic analyses

The average size of the sequenced fragments of *β-tubulin*, *calmodulin* and *ITS* for the 62 studied isolates (Table 2) after editing, were 440, 510 and 550 bases, respectively. A BLAST search on GenBank with obtained *ITS*, *β-tubulin* and *calmodulin* sequences showed high similarity (98–100%) between *P*. *formosus* and our isolates.

**Table 2:** Source of used isolates in phylogeny.

Host	Number of isolates	Isolates
*Pistacia vera*	45	Pf104, Pf107, Pf108, Pf109, Pf111, Pf117, Pf128, Pf130, Pf156, Pf163, Pf233, Pf241, Pf244, Pf250, Pf263, Pf265, Pf285, Pf292, Pf298, Pf310, Pf316, Pf326, Pf346, Pf362, Pf376, Pf396, Pf443, Pf444, Pf450, Pf472, Pf480, Pf515, Pf518, Pf521, Pf559, Pf583, Pf601, Pf613, Pf623, Pf628, Pf659, Pf681, Pf685, Pf694, Pf790
*Pistacia mutica*	2	Pf407, Pf410
*Punica granatum*	3	Pf388, Pf402, Pf475
*Prunus amygdalus*	2	Pf492, Pf780
*Caesalpinia gilliesii*	1	Pf731
*Haloxylon *sp.	1	Pf786
*Nerium oleander*	1	Pf424
*Tamarix aphylla*	1	Pf391
*Tamarix hispida*	3	Pf385, Pf389, Pf774
The air of orchards	3	Pf764, Pf801, Pf804

**Fig 2:**
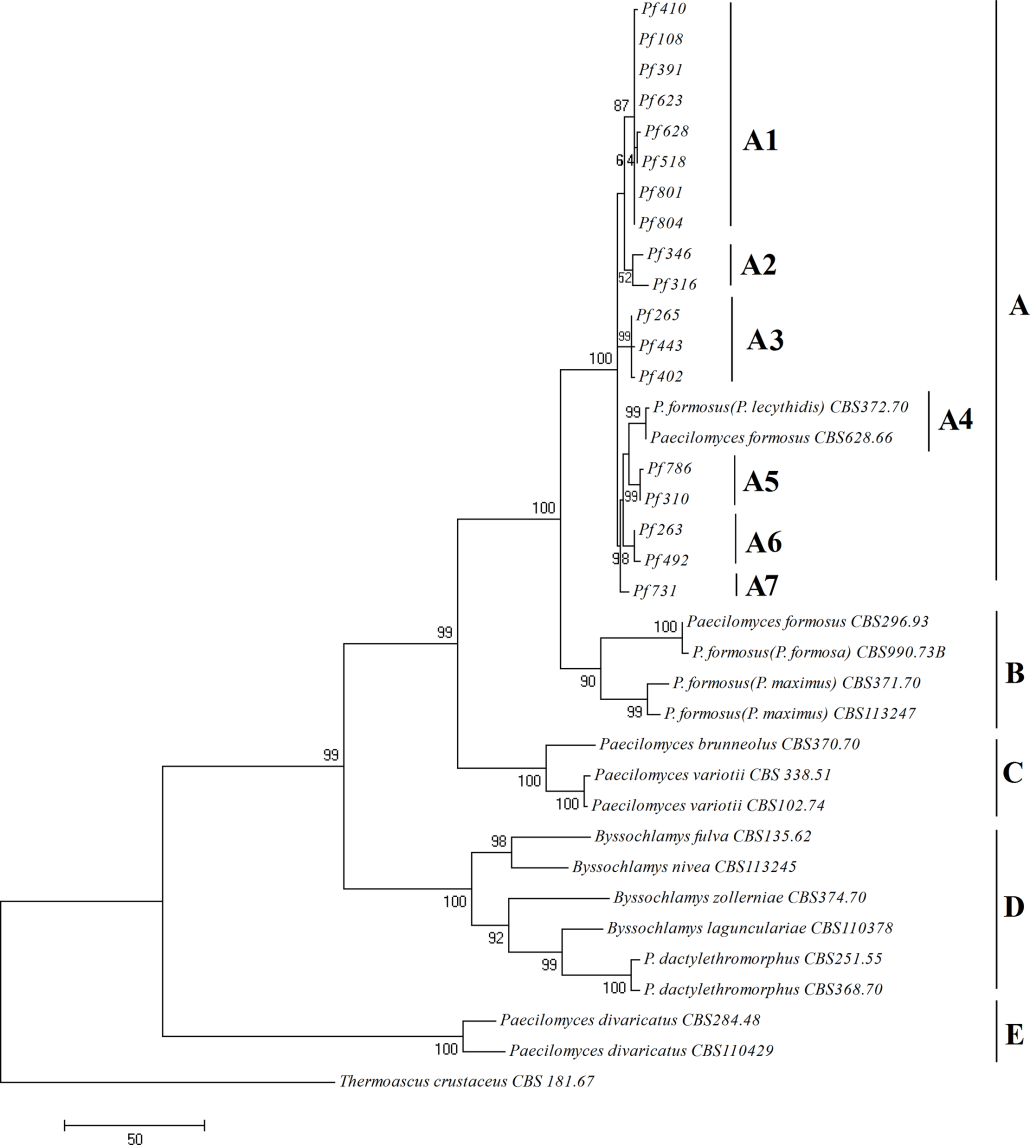
Most parsimonous tree based on combined data set of *ITS*, *β-tubulin* and *calmodulin* sequences. sequences are from 18 selected isolates and close taxa using MEGA 7 software, showing the relationship among our isolates and *Paecilomyces formosus* isolates, especialy to *P*. *lecythidis*. The percentages above and below the branches are the frequencies of a given branch appeared in 1000 bootstrap replications. Bootstrap values less than 50% are not displayed. *Thermoascus crustaceus* has been used as outgroup.

**Fig 3:**
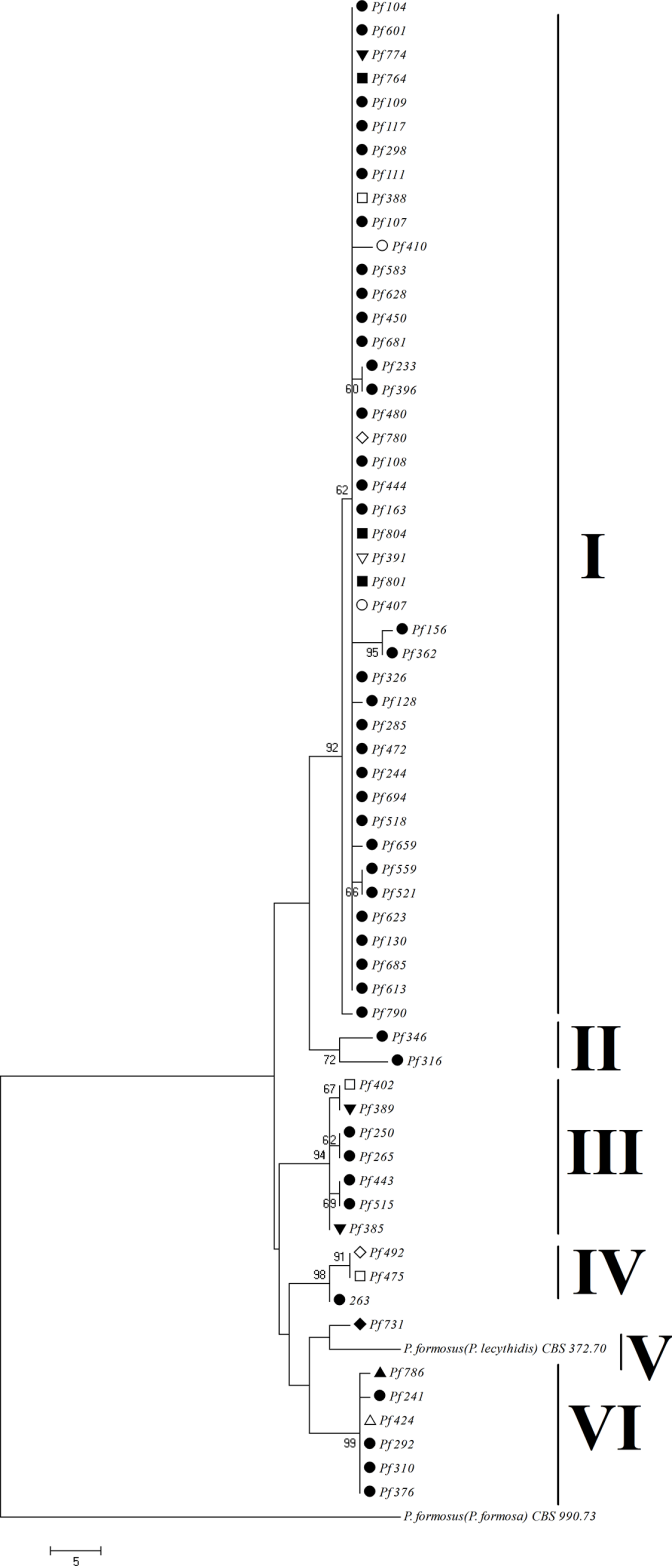
Most parsimonous tree based on combined data set of *ITS*, *β-tubulin* and *calmodulin* sequences. sequences are from 62 selected isolates and close taxa using MEGA 7 software, showing low variation among our isolates. Isolates from *Pistacia vera*, Isolates from *Pistacia mutica*, Isolates from *Punica granatum*, Isolates from *Tamarix hispida*, Isolates from *Tamarix aphylla*, Isolates from *Prunus amygdalus*, Isolates from *Haloxylon* sp., Isolates from *Nerium oleander*, Isolates from *Caesalpinia gilliesii* and Isolates from the air of pistachio orchards. The percentages above and below the branches are the frequencies of a given branch appeared in 1000 bootstrap replications. Bootstrap values less than 50% are not displayed. *Paecilomyces formosus* CBS990.73B has been used as outgroup.

Phylogenetic analyses based on *ITS*, *β-tubulin* and *calmodulin* sequences of our isolates and 18 selected isolates of *Paecilomyces* (Table 3) showed that our isolates are closely related to *P*. *formosus* (Fig 2). All 62 isolates clustered together and formed a well-supported clade with *P*. *formosus* (Fig 3), placed separately from the other species of *Paecilomyces*. Phylogenetic analysis based on combined sequences of 18 selected isolates also confirmed that our isolates form a strongly supported clade (100% bootstrap value) with *P*. *formosus* (clade A in Fig 2). In agreement with physiological studies, *P*. *variotii* isolates belonged to a different clade (clade C) and were different from our isolates. Strains of *P*. *formosus* clustered in three separate groups in sub-clade A4 and clade B supported by high bootstrap values. Six deviating isolates (Pf518, Pf623 and Pf628, which could not grow or produce acid on CREA and should be assigned to *P*. *variotii* based on morphological and physiological features; Pf108, Pf391 and Pf804, which grew less than 45 mm within seven days at 30°C and continuous dark condition on MEA and which could not be identified as any known species), belonged to sub-clade A1 close to *P*. *formosus* and our other isolates and not to the *P*. *variotii* clade (clade C in Fig 2). *Thermoascus crustaceus* has been used as the outgroup.

**Table 3:** Strains and their sequences from GenBank (NCBI) used in phylogenetic analysis.

Species	CBS No.	Gene Bank accession no.			Reference
		*ITS*	*β-tubulin*	*Calmodulin*	
*Byssochlamys nivea*	CBS 113245	FJ389936.1	FJ389998.1	FJ389974.1	[11]
*B. fulva*	CBS 135.62	FJ389943.1	FJ389989.1	FJ389976.1	[11]
*B. lagunculariae*	CBS 110378	FJ389946.1	FJ390006.1	FJ389979.1	[11]
*B. zollerniae*	CBS 374.70	FJ389933.1	FJ390008.1	FJ389966.1	[11]
*Paecilomyces formosus*	CBS 628.66	FJ389927.1	FJ389983.1	FJ389969.1	[11]
	CBS 296.93	FJ389928.1	FJ389994.1	FJ389961.1	[11]
*P. formosus (P. formosa)*	CBS 990.73	FJ389929.1	FJ389993.1	FJ389978.1	[11]
*P. formosus (ex-type strain of P. lecythidis)*	CBS 372.70	FJ389926.1	FJ389990.1	FJ389964.1	[11]
*P. formosus (P. lecythidis)*	DTO 63F1	GU968670	GU968686		[12]
	DTO 63F4	GU968673	GU968688		[12]
	DTO 63E3	GU968664	GU968678		[12]
	DTO 49D6	GU968655	GU968691		[12]
	DTO 45I1	GU968651	GU968684		[12]
*P. formosus (P. maximus)*	CBS 113247	FJ389921.1	FJ390009.1	FJ389980.1	[11]
*P. formosus (ex-type strain of P. maximus)*	CBS 371.70	FJ389920.1	FJ389982.1	FJ389963.1	[11]
*P. variotii*	CBS 102.74	EU037055.1	EU037073.1	EU037038.1	[11]
	CBS 338.51	FJ389930.1	FJ390007.1	FJ389955.1	[11]
*P. brunneolus*	CBS 370.70	EU037050.1	EU037068.1	EU037033.1	[11]
*P. divaricatus*	CBS 110429	FJ389932.1	FJ389991.1	FJ389954.1	[11]
	CBS 284.48	FJ389931.1	FJ389992.1	FJ389953.1	[11]
*P. dactylethromorphus (P. saturatus)*	CBS 368.70	FJ389948.1	FJ390001.1	FJ389972.1	[11]
	CBS 251.55	FJ389951.1	FJ390002.1	FJ389960.1	[11]
*Thermoascus crustaceus*	CBS 181.67	FJ389925.1	FJ389981.1	FJ389952.1	[11]

Maximum parsimony analysis of the combined three sequenced fragments of 62 isolates (Fig 3) could not distinguish isolates of different hosts or different areas except the single isolate Pf731 from *Caesalpinia gilliesii* which belonged to a distinct sub-clade (sub-clade V in Fig 3). Most of the isolates obtained from pistachio, wild pistachio and those from the air of pistachio orchards belonged to subclade I supported by 92% bootstrap value. In other sub-clades some isolates from pistachio clustered with isolates from other hosts (sub-clades, III, IV and VI in Fig 3). Based on the results of the analysis of Fig 3, isolate CBS990.73B (*P*. *formosa*) was chosen as the outgroup (Fig 2). All the sequences were deposited in NCBI Genbank (accession numbers: **MF175886- MF176071**).

## Pathogenicity tests

**Fig 4:**
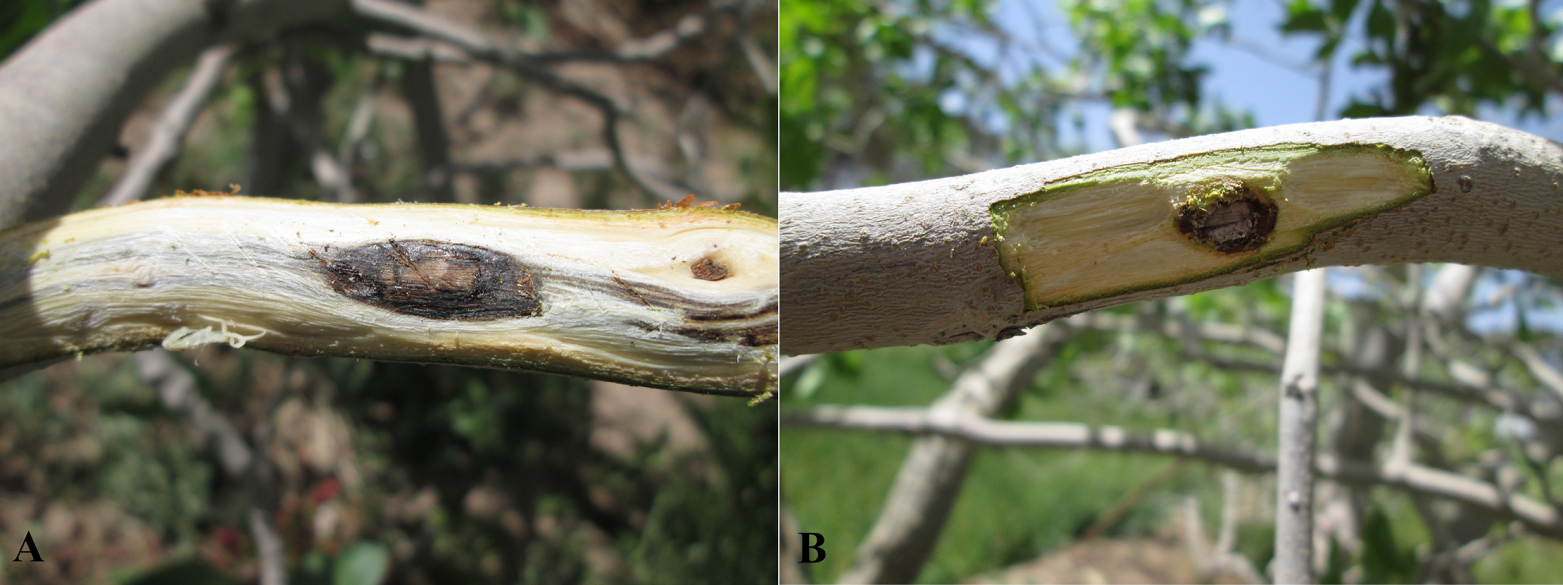
Pathogenicity test. (A) Inoculation of *P*. *formosus* on two years old twigs of pistachio in orchard. Fungus development and canker formation are evident. The fungus develops along the axis of the branch. (B) Control inoculation and callus formation in pathogen free scar.

Pathogenicity tests and Koch's postulates were performed and causative relationship between isolates and dieback disease was confirmed. Cankers were developed on healthy twigs of host trees in orchard (Fig 4)

## Discussion

Pistachio is an important export product of Iran. Pistachio dieback is a significant disease in 400,000 hectares of Iranian pistachio orchards. In our samplings from 10 provinces, the symptoms of dieback disease were observed in at least 85% of pistachio orchards. However, Alizadeh et al. [3] had observed symptoms of dieback in 17% of pistachio orchards in Rafsanjan area in Kerman Province. The increase of infection in pistachio orchards shows that several conditions have predisposed pistachio trees to pathogen distribution and disease prevalence. About 1–100% of pistachio plants in affected orchards were showing symptoms of dieback and 1–100% of branches and twigs in infected trees had been injured by dieback disease. In some areas, development of canker on aerial parts of pistachio trees had decreased the size of canopy to half of that of healthy trees. Of the 567 infected pistachio branch and twig samples, 277 *Paecilomyces* strains (49%) were obtained. Several isolates of *Cytospora*, *Neofusicoccum*, *Coniothyrium*, *Ulocladium*, *Alternaria*, *Fusarium*, *Cladosporium*, *Aspergillus* and *Penicillium* also were isolated from collected samples. Alizadeh et al. [3] tested pathogenicity of *Paecilomyces variotii*, *Neofusicoccum mangiferae* (*Nattrassia mangiferae*) and *Cytospora* sp. on different cultivars of pistachio in Rafsanjan and suggested that the other recovered fungi (*Coniothyrium*, *Ulocladium*, *Alternaria*, *Fusarium*, *Cladosporium*, *Aspergillus* and *Penicillium*) were saprophytes. This disease also was observed on other trees in pistachio growing areas and we collected 133 infected branch and twig samples which were from 27 plant species and finally 23 (about 17%) isolates of the genus *Paecilomyces* were obtained from only nine plant species including *Pistacia mutica*, *Punica granatum*, *Prunus amygdalus*, *Caesalpinia gilliesii*, *Nerium oleander*, *Tamarix aphylla*, *Tamarix hispida* and *Haloxylon* sp. Only *Paecilomyces variotii* had been reported as dieback agent on pistachio [3] and almond [16] in Iran previously. The samples tested in this study were collected from Kerman, Yazd, Isfahan, Qom, Markazi, Qazvin, Tehran, Semnan, South Khorasan and Khorasan Razavi provinces which have different climates, ranging from mountainous, temperate, sub-tropical to hot and dry, and pistachio could grow in all of these climates but most of pistachio growing areas are in hot and dry conditions in the provinces mentioned, although no considerable differences were observed in distribution and prevalence of disease between different climates.

The Kerman Province is the major producer of pistachio in Iran. The economical pistachio production in Kerman province is estimated to be less than 300 years old, but *Pistacia mutica*, *Punica granatum*, *Tamarix aphylla* and *Tamarix hispida* are native plants and older than pistachio. The pattern of host specificity (Fig 3) suggests that this pathogen was first parasitic on native trees and subsequently shifted to pistachio trees. The almond is cultivated in mountains in Iran and maybe this pathogen has shifted to almond from *Pistacia mutica* which is grown wildly in mountains. *Caesalpinia gilliesii* and *Nerium oleander* are ornamental plants and *Haloxylon* spp. are type of trees that are tolerant to harsh conditions of deserts and are used for reforestation the desert in Iran. The disease could be transferred to these three plant species from native hosts or pistachio. Five isolates including Pf764, Pf799, Pf801, Pf802 and Pf804 obtained from the air of infected pistachio orchards in late fall and early winter a week after raining, on other times isolates were not obtained from the air. The pathogen was alive in cankers of infected trees throughout the year. Since the pruning of pistachio is done in late fall and early winter, the results of this study show that most of new infections taking place in late fall and early winter and pathogen is transferred via the air and pruning equipments (in agreement with Alizadeh et al. [3], Aminaei and Ershad [4] Ghelichi et al. [5] and Mohammadi and Haghdel [2]).

Most of the strains which we isolated from *Pistacia vera* and the air of pistachio orchards were placed in clade I (Fig 3), Only four isolates came from other tree species (*Tamarix hispida*, *Punica granatum* and *Prunus amygdalus*, and the wild relative of *Pistacia vera*, the *Pistacia mutica*). This pattern shows that a single clade mostly consisting of a single haplotype, has specialized on *Pistacia vera*. We found that maximum parsimony analysis of the *ITS*, *β-tubulin* and *calmodulin* sequences and combined three sequenced fragments could not distinguish isolates of different hosts or different areas.

Until now, all researchers in Iran have used the morphological features for the identification of *Paecilomyces* species, therefore *P*. *variotii* has been reported as causal agent of dieback disease on pistachio and almond, but morphological characters alone are not sufficient for species identification. *P*. *variotii* is a species complex consisting of five species which could be divided by physiological and phylogenetic criteria [11]; therefore, utilization of both physiological and phylogenetic analyses was necessary for pathogen identification. Morphological examinations classified all the isolates in *P*. *variotii* sensu lato in agreement with previous morphological studies [2–5]. Physiological tests including growth rate at different temperatures on MEA, CYA and CREA and acid production, showed that 299 of 305 isolates (98%) belong to *P*. *formosus* based on Samson et al. [11]. Three isolates, Pf518, Pf623 and Pf628 which grew rapidly on MEA attaining a diameter more than 45 mm within seven days at 30°C and continuous dark condition, did not grow and produce acid on CREA medium and should be assigned to *P*. *variotii*. Three strains with slow growth (less than 45mm per week; isolates Pf108, Pf391 and Pf804) which grew and produced acid on CREA medium could not be assigned to any species based on Samson et al. [11]. Therefore, morphological and physiological investigations assigned 98% of isolates to *P*. *formosus*, 1% to *P*. *variotii* and 1% could not be linked to any known species. Phylogenetic analyses based on DNA sequence data and comparing isolates with known species showed that representatives of all 305 isolates belong to *P*. *formosus*, six physiological different isolates (Pf108, Pf391, Pf518, Pf623, Pf628 and Pf804) also clustered together with our other isolates in the *P*. *formosus* clade (clade A1 in Fig 2 and clade I in Fig 3). Physiological and phylogenetic studies had a high level of agreement (98%), because phylogenetic tests assigned 100% and physiological tests 98% of isolates to *P*. *formosus* but 2% of isolates were different physiologically. The primary concept of Samson et al. [11] for dividing *P*. *variotii* sensu lato to five species was based on a molecular phylogeny, after which they compared strains and described physiological characters (growth rate at different temperatures on special culture media and acid production) as differential features for identifying species. We hypothesize that 2% inconsistency between phylogeny and physiology arises from a mutation or gene silencing or the rate of acid production is lower than medium sensitivity. The isolates showed some variations in shape, color and reverse of colonies which maybe some intra-specific variations and are not useful for species identification.

In phylogenetic trees based on *ITS* and *β-tubulin* sequences data, some of recognized strains of *P*. *formosus* including CBS 372.70, DTO63F1, DTO63F4, DTO63E3, DTO49D6 and DTO45I1 were located in our isolates clade, although some of them including CBS990.73, CBS113247 and CBS 371.70 were grouped as a sister group of our isolates clade. Therefore, we reinvestigated them and revealed that the isolates which were grouped in our isolates clade, had been classified in a former species *P*. *lecythidis* and other strains which were grouped in sister group had been classified in former species *P*. *formosa* and *P*. *maximus* (Table 3). As a result of this study and according to the studies by Houbraken et al. [12] and Samson et al. [11] it seems that *P*. *formosus* is a species complex, consisting of three taxa, *P*. *formosus*, *P*. *lecythidis* and *P*. *maximus*. However, the basis of this concept is phylogeny and the three taxa could not be identified by microscopic examination.

Maximum parsimony analysis of combined data set of *ITS*, *β-tubulin* and *calmodulin* sequences showed low variation between isolates (<1%), although we had isolated our samples from different areas, hosts and varieties. This shows that our isolates all descend from a recent common ancestor.

Finally, the causal agent of die-back disease of pistachio and other examined trees is *P*. *formosus* which is closely related to former species *P*. *lecythidis* and has some differences with former species *P*. *formosa* and *P*. *maximus*.

## Material and methods

### Samples

In the fall and winter of 2012, the winter, spring, summer and fall of 2013 and the winter and spring of 2014, 700 plant samples from infected branches and twigs of pistachio and other trees with dieback symptoms were collected from private orchards located in 10 provinces of Iran, where the pistachio trees are cultivated (Table 1). The infected plant samples were taken with permission of the orchard owners. These provinces are Kerman, Yazd, Isfahan, Qom, Markazi, Qazvin, Tehran, Semnan, South Khorasan and Khorasan Razavi. The samples were collected from most of the important pistachio varieties (Fandoghi, Kalleghoochi, Ahmadaghaii, Akbari, Badami, Khanjari, Abbasali, Shahpasand and non-grafting) and different environmental conditions such as mountainous, temperate, sub-tropical and hot and dry.

Ethics Statement: We confirm that the field studies did not involve endangered or protected species and no specific permission was required for sampling.

### Fungal isolates

The infected plant samples were washed with tap water for 10 minutes and then dried on sterile tissue paper, upon which 6 slant sections (5×5mm) from the border between infected and healthy tissues were cut. The sections were surface sterilized in 0.5% sodium hypochlorite for 1–2 minutes, floated in sterile water for 24 hours to eliminate phenolic compounds and deterrents, then dried on sterile tissue paper, transferred to potato dextrose agar (PDA) plates and incubated at 25°C in continuous dark condition. After seven days, plates were inspected and *Paecilomyces* colonies were transferred to water agar (WA) plates and incubated at 25°C for seven days in continuous dark condition. Pure fungal colonies were obtained with the hyphal-tip technique. Pure isolates were cultured on MEA plates. After the colony covered the plates, one gram of MEA including mycelia, conidia and chlamydospores were placed in 1.5 ml vials and stored at 4°C.

### Morphological and physiological studies

The culture media used for macro- micro-morphological and physiological studies were include MEA, CYA and CREA. Culture media compositions were according to the Frisvad and Samson [17]. Isolates which had been cultured on MEA and CYA were incubated at 30 and 37°C for seven days in continuous dark condition. For micro-morphological observations of the asexual state, microscopic slides were made in lactophenol and lactophenol-cotton blue from MEA colonies, incubated at 25°C. The size of 100 conidia, conidiophores, phialides and chlamydospores were measured. CREA is a semi-selective culture medium, which can be used for dividing closely related species based on rate of colony growth and acid production at 25°C for seven days in continuous dark condition (when an isolate produces acid, the color of the culture medium is turning from purple to yellow). Colony diameter in seven days on MEA and CYA at 30 and 37°C was recorded. Growth pattern and colonies back color were recorded. Results of morphological and physiological studies were compared with Samson [8] and Samson et al. [11].

### Phylogenetic analyses

62 isolates were selected based on the morphology, physiology, host characteristics and geography for a phylogenetic analysis. The isolates were grown on MEA at 25°C for 3 to 4 days. Total fungal genomic DNA was extracted from the mycelium according to Liu et al. [18], and then quantified using a NanoDrop (NanoDrop 2000, Thermo Scientific, USA). The *ITS* region (including the internal transcribed spacer regions 1 and 2, and the 5.8S rRNA regions of the nuclear ribosomal RNA gene cluster) was amplified using primers ITS1 and ITS4 [19]. Amplification of part of the *β-tubulin* gene was performed using the primers Bt2a and Bt2b [20]. A fragment of the *calmodulin* gene sequences was amplified using the primers cmd5 and cmd6 [21]. Excess primers and dNTPs were removed from the PCR product using the Genelute PCR clean-up kit (Sigma Aldrich, USA). Purified PCR fragments were quantified using a NanoDrop, then sequenced by Eurofins Company (Ebersberg, Germany). The electropherograms of the nucleotide sequences were inspected and edited using ChromasPro (version 1.7.6, Technelysium, Australia). A BLAST search with the *ITS*, *β-tubulin* and *calmodulin* sequences was done to show the relationship of the sequences with the ones deposited in Genbank. For phylogenetic analysis, *ITS*, *β-tubulin* and *calmodulin* sequences of closely related species were obtained from GenBank (Table 3) and all sequences were aligned with the same sequences of our *Paecilomyces* isolates using the MAFFT 7.182 software (standard settings, EMBL-EBI, UK). After alignment, the nucleotide data were used in the phylogenetic analysis in the MEGA 7 package (Molecular Evolutionary Genetics Analysis, Biodesign Institute, USA). Both neighbor-joining (NJ) [22] and maximum parsimony (MP) [23] methods were used for phylogenetic reconstruction. Maximum parsimony analysis was performed with the heuristic search option. To assess branch support values, bootstrapping [24] was done, using 1000 replications. Alignment gaps were treated as missing data and all characters were unordered and of equal weight. The species *Thermoascus crustaceus* CBS 181.67 was used as outgroup taxon. Phylogenetic trees obtained by maximum parsimony analysis of each of the datasets of *β-tubulin*, *calmodulin* and *ITS* exhibited the same topologies and our isolates clustered together in a distinct and well-resolved clade. However, the phylogenetic relationships of a few isolates differed slightly between the three phylogenetic trees, so that we selected 18 isolates from the different sub-clades of the trees in each fragment and combined the three different sequences of each isolate and closely related species from GenBank, and then made a single alignment (the P value was 0/1–1) and performed a maximum parsimony analysis for the three datasets using the heuristic search option. Finally, a maximum parsimony analysis was performed for the combined three sequences of all our isolates to study the relationship between isolates of different hosts from different areas. *Paecilomyces formosus* CBS 990.73B which showed some differences from our isolates, was used as the outgroup for this analysis.

## Pathogenicity tests

Previously, pathogenicity of several isolates of *Paecilomyces* was confirmed in pistachio and almond [3,5,16] in vitro and in situ, but the causal agent has been introduced as *Paecilomyces variotii*. Therefore, we performed the pathogenicity tests and Koch's postulates to confirm the causative relationship between isolates and dieback disease in hosts. The fungal strains were inoculated to the hosts that were isolated from them. For *Pistacia vera*, *Punica granatum*, *Prunus amygdalus*, *Tamarix hispida* and *Haloxylon* sp., three isolates and for *Pistacia mutica*, *Tamarix aphylla*, *Caesalpinia gilliesii* and *Nerium oleander* respectively 2, 2, 1 and 1 isolates were inoculated on healthy twigs of host trees in Iranian Pistachio Research Institute, Rafsanjan, Iran orchard with three replicates and three controls and with the permission of head of the Institute. Two isolates which were isolated from the air, were inoculated to *pistacia vera*. Twigs were investigated after 30 days, if the canker had been formed, and then the fungi were re-isolated and identified.
